# Privacy and data protection in learning analytics should be motivated by an educational maxim—towards a proposal

**DOI:** 10.1186/s41039-018-0086-8

**Published:** 2018-12-11

**Authors:** Tore Hoel, Weiqin Chen

**Affiliations:** Oslo Metropolitan University, Postboks 4 St. Olavs plass, 0130 Oslo, Norway

**Keywords:** Privacy, Data protection, Learning analytics, Data privacy

## Abstract

Privacy and data protection are a major stumbling blocks for a data-driven educational future. Privacy policies are based on legal regulations, which in turn get their justification from political, cultural, economical and other kinds of discourses. Applied to learning analytics, do these policies also need a pedagogical grounding? This paper is based on an actual conundrum in developing a technical specification on privacy and data protection for learning analytics for an international standardisation organisation. Legal arguments vary a lot around the world, and seeking ontological arguments for privacy does not necessarily lead to a universal acclaim of safeguarding the learner meeting the new data-driven practices in education. Maybe it would be easier to build consensus around educational values, but is it possible to do so?

This paper explores the legal and cultural contexts that make it a challenge to define universal principles for privacy and data protection. If not universal principles, consent could be the point of departure for assuring privacy? In education, this is not necessarily the case as consent will be balanced by organisations’ legitimate interests and contract. The different justifications for privacy, the legal obligation to separate analysis from intervention, and the way learning and teaching works makes it necessary to argue data privacy from a pedagogical perspective. The paper concludes with three principles that are proposed to inform an educational maxim for privacy and data protection in learning analytics.

## Introduction

A data-driven educational future has to navigate the stumbling blocks of privacy and data protection. Educationalists often find that dealing with these thorny issues are the prerogative of other professions such as lawyers or computer scientists and that pedagogical perspectives are not represented in the discourse. In preparing for the digital futures of learning analytics (LA), adaptive education, multimodal learning support and other data-driven approaches educationalists need to develop what we have termed an educational maxim for privacy and data protection in this field.

Privacy and data protection measures are often promoted and justified by laws and regulations. Two recent events have created international awareness about the importance of privacy, the ‘Facebook–Cambridge Analytica data scandal’ (n.d.), and the General Data Protection Regulation (GDPR) of the European Union (European Commission 2016), which came into effect in May 2018. The first event prompted social media users to ask themselves about their own data sharing practice; while the GDPR prompted most companies, also outside of Europe, to revisit their data protection rules in order to avoid huge economic penalties in case of data breaches.

The discussion on privacy we now see spurred by these events is just the pinnacle of more than 50 years of international debate on privacy. In the USA, in the 1960s, ‘privacy’ was invoked as a key term for summing up ‘the congeries of fears raised by the (mis)use of computers’ (Bygrave 2010). Privacy was not the only term; a ‘variety of other, partly overlapping concepts have been invoked too, particularly those of ‘freedom’, ‘liberty’ and ‘autonomy’ (ibid., p. 167). Today, in other parts of the world the backdrop of privacy may be less that of liberal values than of avoiding loss of face in privacy breach scandals (as an example, see the news story ‘Jack Ma’s Ant Apologizes for Baiting Users Into Credit System’ (Chen 2018)). When raising the discussion of privacy and data protection in a new context—i.e. learning analytics—we have to factor in the very complex global data protection scene where legal regulations are debated on a background of diverse political, cultural, economical and even philosophical ideas.

The question raised in this paper, is whether there also are pedagogical ideas that should be brought to bear when designing privacy policies and solutions for educational big data. For example, are there educational specific requirements that will justify a practice that goes beyond what is required by law? If this *extra* requirement is found, it should ideally be summarised in an educational maxim that ideally would resonate well enough to bridge some of the gaps we find between different legislations and cultures related to how privacy is valued or conceptualised.

This paper aims at exploring the grounds for this educational ‘extra’ that would allow us to be bold in involving the students in self-managing their own data used for learning analytics. We do this exploration on the backdrop of a heterogeneous international landscape regarding the rights of the individual and the value of privacy. To construct the foundation for an educational maxim for privacy related to educational big data this conceptual analysis builds on issues and concerns identified in design of LA applications. Subject of scrutiny will be different positions on how privacy may be invoked and promoted in technology enhanced learning in different international settings. The questions leading up to the proposal of grounding privacy for LA in an educational maxim are the following:

- Is reference to privacy as an individual universal right the answer to data management and control?
- Is consent the mechanism to use to get access to data?
- Is maintaining privacy a question of negotiation, and if so,
- What are the opportunities for pedagogical reasoning and justification of certain privacy related practices?

These questions describe the structure of this paper, which concludes with three principles that could be used to further develop an educational maxim for data privacy in learning analytics. In the next section, we give a practical context for why this research needs to be undertaken.

## Research background through a practical case

This section brings a snapshot of development of an ISO standard on Privacy. ISO/IEC JTC1/SC36, the ISO committee working on interoperability standards for learning, education and training has a working group 8 focusing on learning analytics. In the first working draft of a new technical specification on privacy and data protection, it was admitted that privacy is difficult to define restrictively ‘as privacy is an elusive concept that means different things in different countries around the world. What is seen as an intrusion into the private life or affairs of an individual, and whether gathering of data about the individual is seen as undue or illegal varies with cultural context’ (T. Hoel, personal communication, August 2018). The editors of the draft specification suggested that privacy problems should be looked at ‘in a LET [learning, education and training] context to be able to specify privacy and data protection principles for LET that address specific problems and support a good learning environment for the individuals involved’. The editors suggested the following principle for development:

‘The educational context of LA requires that the right to be informed is not interpreted restrictively; it is a pedagogical value of its own to be as open as possible about data collection and processing.’

And regarding the legal requirements of notification of the data subject of data collection, the first working draft stated:

‘Age of the students, the educational setting, matters of authority, and other reasons could influence how notification of data collection and processing will be conceived. The educational context is, however, an opportunity to clarify [for the students] privacy and data protection issues related to use of LA’ (T. Hoel, personal communication, August 2018).

From this working draft, it is clear that the authors of the standard try to carve out an educational argumentative space that would allow for certain policy principles regarding privacy. In this space, one finds arguments about involvement of students, openness, and what we could term educational opportunity (‘you should teach about big data, data management, and privacy – here you have an opportunity to do so’) (Pangrazio and Selwyn 2018).

## A universal right to privacy using educational technologies?

Even if educational policies often are the purview of local authorities, when we talk about educational technologies—like LA—we are dealing with global solutions that have to cater for all political and cultural climates. In this section, we examine privacy and data protection in an international perspective, starting by asking if there is a universal right to privacy.

Milberg et al. (1995) stated that it could be reasonably argued that protection of personal information privacy was a ‘hypernorm’, a principle fundamental to human existence. ‘If this is so, then managers have an obligation to protect personal information privacy in every system and in every country, regardless of distinctions in national levels of concern or of regulatory approaches’ (Milberg et al. 1995, p. 73). However, research on the relationships among nationality, cultural values, personal information privacy concerns and information privacy regulations led Milberg et al. (1995) to conclude on a more pragmatic note: ‘Executives may choose to reject the ethical “hypernorm” argument (…) But the threat of negative impacts on the bottom line, driven by both market forces and the legislative agenda, should be sufficient to prod them towards a more enlightened view of the personal information privacy management domain’ (p. 73).

Further research by Milberg et al. (2000) found that most firms took a primarily reactive approach to managing privacy ‘by waiting for an external threat before crafting cohesive policies that confront their information practices’ (p. 49).

When ideals meet stakeholders’ interests trade-offs are inevitable. Milberg et al. (2000) find that ‘[a] right to privacy’ has been taken to include a number of ‘interests’ that converge and diverge, and they use targeted marketing as an example of trade-offs between the privacy interests and how society’s economic and social systems function:

‘While organizations argue that they have the right to conduct business, consumers and privacy advocates often claim the right to be free of unwanted solicitations. While organizations claim the right to use information technology to improve efficiency, consumers often exhibit the desire to control the flow and dissemination of their personal information. While businesses claim the right to record information generated from their transactions, consumers increasingly want to know that this information has been gathered and stored and to control its uses’ (Milberg et al. 2000, p. 36).

Trade-offs between ideals and reality may not be the best way to understand how privacy and related interests with regard to the processing of personal data are protected internationally. Alternatively, one could see how these issues are conceptualised in different countries, and how the different discourses express values that are taken up by different regulatory policies. A full analysis of this kind is beyond the remit of this paper. However, a study by Bygrave (2010) exploring the prospects for regulatory consensus found that data protection laws in various countries ‘expound broadly similar core principles and share much common ground in terms of enforcement patterns’ (Bygrave 2010, p. 198). Nevertheless, ‘extensive harmonisation at the global level is extremely unlikely to occur in the near future’ (ibid., p. 199). The reason for this lack of harmonisations is the strength of ‘ingrained ideological/cultural differences’ (ibid., p. 199).

Even if extensive harmonisation of international privacy laws is hard, and a number of countries lack such laws all together, there is a global trend towards privacy legislation due to the growing impact of the digital economy. Table 1 shows the status of data protection laws in ten Asian countries. Six of them have data protection laws that have been amended recently; the other four countries have plans to pass laws or address privacy issues in closely related laws (as China).

**Table 1:** Status data protection laws in some Asian countries (Primary source: DLA Piper 2017)

Country	Data protection law?	Future plans
China	No	No comprehensive data protection law. However, Cybersecurity Law (2017) first national-level law that addresses cybersecurity and data privacy protection.
India	No	Draft Personal Data Protection Bill published 2018
Indonesia	No	Draft personal data protection law published 2018.
Japan	Yes (2017)	
Malaysia	Yes (2013)	
Philippines	Yes (2012)	
Singapore	Yes, only private sector (2012)	
Thailand	No	Draft is being reviewed (as of 2016).
Taiwan	Yes (2012)	
South Korea	Yes (2011)	

Whom should privacy serve? Even if privacy legislation around the world draws on common ideas and principles, there are clear differences in the way privacy is conceptualised. In the USA, most discourse on privacy and privacy rights tends ‘to focus only on the benefits these have for individuals *qua* individuals’ (Bygrave 2010, p. 171) (…) while German jurisprudence ‘emphasises that the value of data protection norms lies to a large degree in their ability to secure the necessary conditions for active citizen participation in public life; in other words, to secure a flourishing democracy’ (ibid., p. 172). While Germany has had the most comprehensive and well-established legislative platform for data protection, USA has had an absence of comprehensive data protection legislation. Germany has had to harmonise with the other European countries after GDPR came into effect. Globally, it is expected that GDPR will have an influence on future legislation in countries also outside of Europe. One example is India, where the draft (MEITY 2018) clearly mimics some of the features of GDPR (e.g. the principles of purpose and collection limitations, privacy by design), but stops short of EU’s privacy-safeguarding regulations in the matter of individual’s right to object to collection and/or processing of their personal information.

The formal normative basis for the data protection laws may well be derived ‘mainly from the catalogues of fundamental human rights’ (Bygrave 2010, p. 180); however, when it comes to applying these principles in international instruments one should note that an important motivation for developing international privacy frameworks are promoting the exchange of goods and services across borders. Bygrave (2010) claims that in the Asian Pacific region, the approach ‘appears to foster data protection regimes less because of concern to protect basic human rights than concern to engender consumer confidence in business’ (Bygrave 2010, p. 188).

**Fig. 1:**
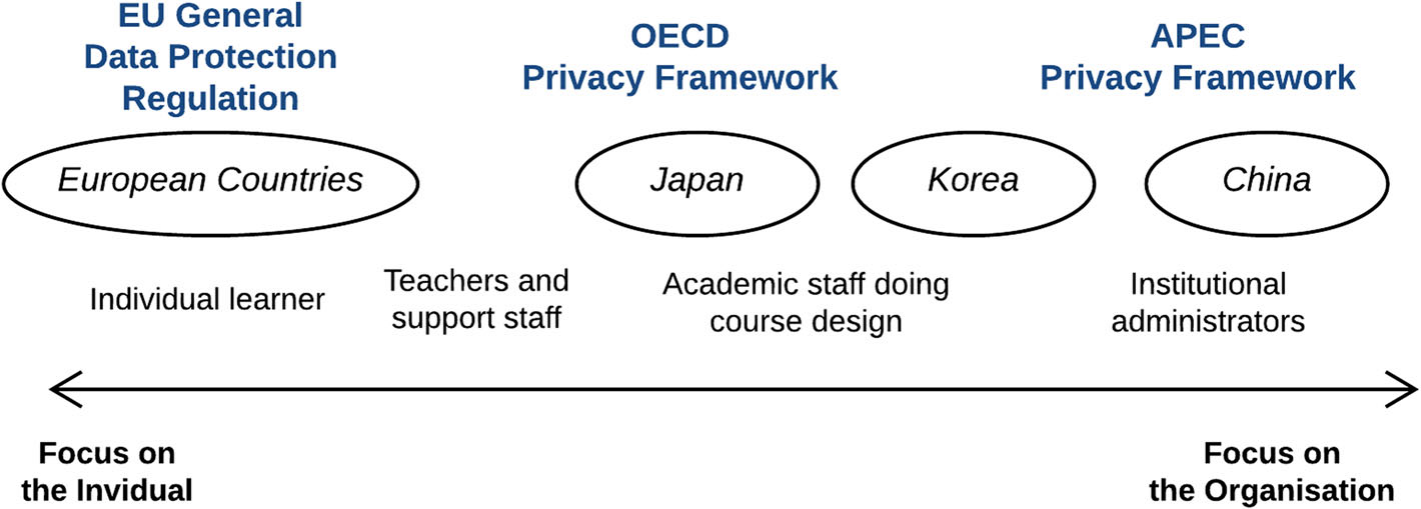
Individual vs. organisational focus of LA beneficiaries, privacy frameworks and countries

Hoel et al. (2017b) analysed three privacy frameworks, which have inspired legal development in all parts of the world and put the frameworks and selected countries on a scale with values between a focus on the individual and a focus on the organisation (Fig. 1).

The case studies of the LA privacy discourse in Europe and Asia (Japan, Korea and China) (Hoel et al. 2017) showed that concerns about the rights of the individual in relation to control of data emanating from the learner are in some respect a western phenomenon. In the east, where the interests of the individual more often are projected against the interest of the group the organisation is more prominent in the discourse on who should benefit from LA.

In this section, we have seen that even if the concern for data privacy is shared among the general public around the world there is a long way to go from concern, at least in the abstract, to finding a common normative basis for establishing data protection policies. The global ideological landscape does not invite to subscription of human rights ideas or other shared normative ethics principles to motivate regulatory consensus on data protection. Lately, both societal and individual arguments have made the discussion on privacy more complex. War on terror, national security, promotion of trade and new digital economies are all factors that demand extensive sharing of personal data. We also see that the users of ICT services are willing to undermine their own rights as soon as they see short time benefits of opening up access to their personal data (Hazari and Brown 2014). In the next sections, we will explore how involvement of the individual could be used to justify data sharing.

## Educational data privacy by asking for consent

‘Obtaining valid consent from data subjects in connection with the use of personal data for analysis and profiling purposes is the best insurance against violating data protection legislation. The new European Data Protection Regulation also proposes restricting the opportunities for the processing of personal data on legal grounds other than consent’ (Datatilsynet 2013, p. 49).

We find it interesting that the Norwegian Data Protection Authority uses the phrase ‘best insurance’ in their 2013 report *Big Data - privacy principles under pressure*. Risk minimisation is the word of the day now as industry and public organisations alike for some time, under threats of heavy fines, have prepared for compliance with GDPR, setting up accountability systems, documenting what information one holds, assigning data protection officers, and taking other organisational measures. However, risk management is a different strategy than invoking rights, and such a strategy certainly chooses the organisational perspective, as opposed to the individual perspective that comes with arguing from rights. So, what does it mean when the Norwegian Data Protection Authority states as their primary recommendation to meet the challenges of big data: ‘consent [is] still the point of departure’ (Datatilsynet 2013)? Is consent, *in the context of LA*, the primary point of departure?

Focussing on consent means bringing the individual into the centre of the discussion. And that means the individual as an actor with rights to decide on data management, not as an object in need of protection by others. However, consent in the age of Big Data is not straight forward. The Norwegian data protection authority points to claims, ‘that the constant demand for consent on the Internet paradoxically may result in poorer protection for the individuals’ (ibid, p. 50). Now, with the new GDPR asking for wide-ranging consent it may be a lesser problem if you live in a European country. The new Regulation has strengthened the protection from giving your rights away by ticking boxes when launching software solutions. The problem with consent, we would argue, does not so much lie in hollowing out the consent mechanism as with the fact that consent is not the sole legal ground for access to personal data. And pretending it is, will confuse the individual and undermine the individual’s ability to manage one’s own data.

In an educational setting, there are a number of stakeholders with legitimate rights to a person’s data, driven by the fact that the student has an obligation to go to school or has registered for a course, and in practice entered a contractual relation with an (business) organisation. It is not clear cut what the legal grounds for access to data are; let us say for an administrator, a teacher, or a third party. Data about a student starts to build up from the moment the student does a web search in the course catalogue, right up to the clicks made browsing through learning resources, passing tests, and getting an exam. An educational institution is a business organisation with student records, which are not under the full control of the students. Nobody will contest that right of the institution to store and analyse data about who is registered for what course, and who ends up with what exam results. But what about the results of micro tests? There are no clear boundaries between data generated that are solely the student’s prerogative to manage, and data that the institution, the teacher, has a right to process (Zeide 2017). These are issues that are subject to negotiations between parties that will base their positions on both legal, moral and pedagogical grounds.

**Fig. 2:**
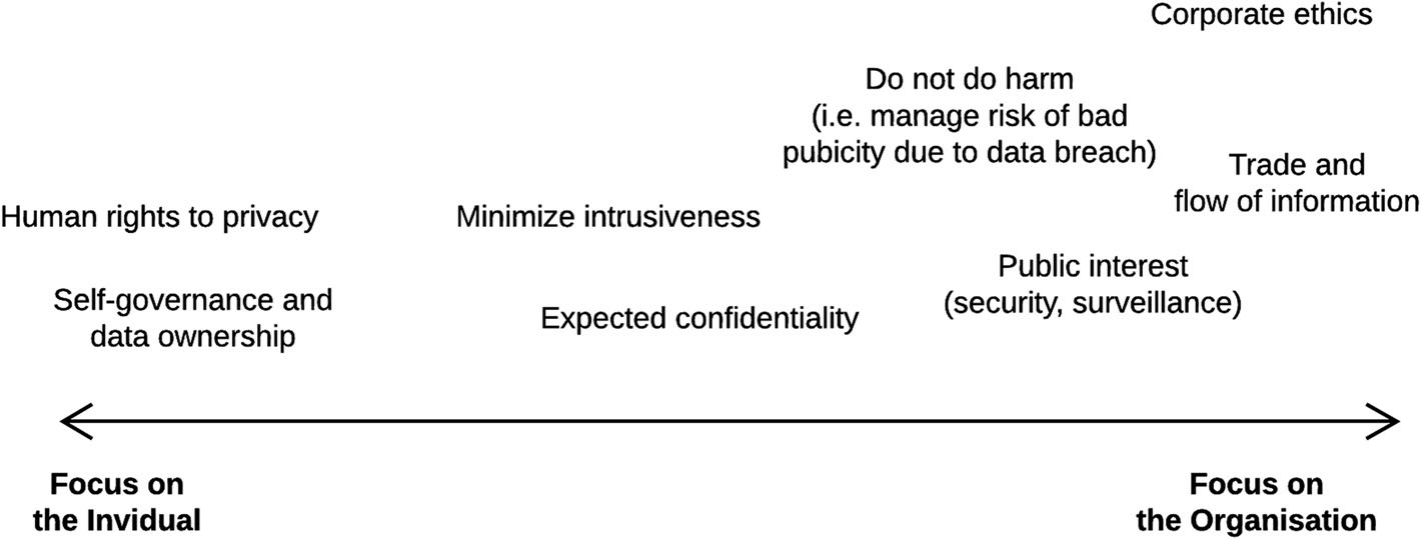
Normative basis for privacy policies

We asked if consent is the primary point of departure within an educational context, and we have answered no. If we overlay the discussion in this section with the observations made in section 3 on the normative basis for privacy in a global perspective (Fig. 2) we see that the role given to consent (and to the individual) could vary a lot in different cultural, political, legal and regional contexts. There is a need to explore more in detail how different scenarios will play out for consent from learners in connection with use of personal data for learning analytics. And we also see there is a need to explore the educational perspective on data privacy.

## Balancing interests for educational big data analysis

Once leaving the abstract reflection on privacy and entering the field of practical data handling we see that the context and the purpose of data collection are important for how data privacy should be handled. As an example, let us compare how equally sensitive personal data gathered from passing through an airport, visiting a hospital, and taking part in education are handled. Public interests will trump any objection from the individual to be scanned by security cameras in the airport. In contrast, in a hospital, the individual has an absolute right to be a party to the data processing, and in extreme cases have the right to refuse to be given lifesaving treatment. Health and education are quite similar in that the individual is very much ‘part of the treatment’, and therefore consent should be sought. However, there are differences. Some education is compulsory. If consent is not the justification for processing personal data, there must be another, e.g. contract, legal obligations, vital interests of the organisation, public interest, or legitimate interest of the data controller.

**Fig. 3:**
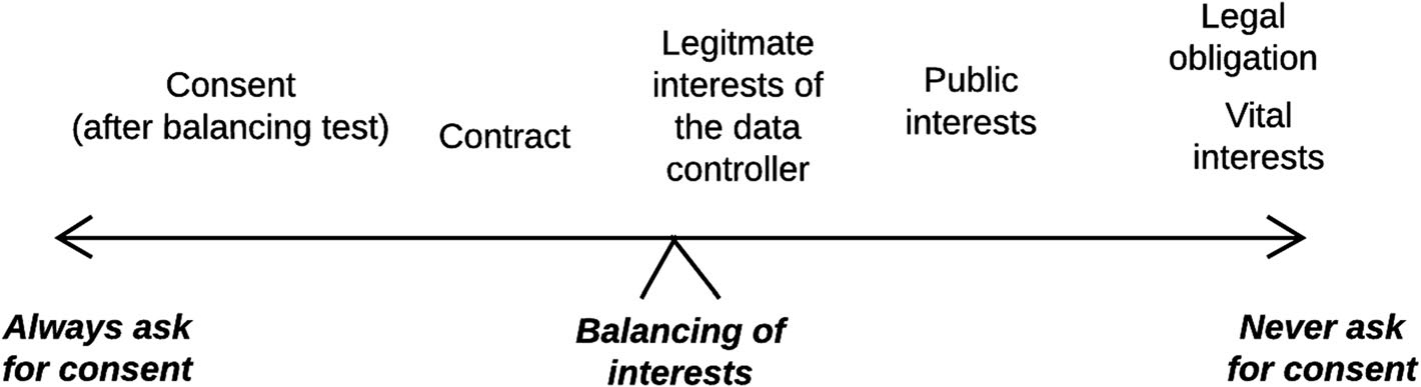
Balancing of interests, asking for consent to process personal data

Figure 3 describes how a decision to ask for consent is a balancing act weighing different interests considering the different justifications for collecting and processing personal data.

In education, especially in the new data-driven practices involving use of online platforms and sensor data, we do not think the data controller will be justified *never* to ask for consent invoking legal obligation or vital interests (the right side of the continuum in Fig. 3). Contract or legitimate interests (e.g. business reasons) on the other hand, would be convenient to invoke, to allow data collection and processing without too much interference of the individual. However, if demands from the students to be involved are getting strong also business reasons will drive the balance to the left in Fig. 3.

We would assume that educational institutions will justify their data processing either by consent or legitimate interests, e.g. stated in a contract. What are the limits to using legitimate interests, and are there any reasons related to LA that would speak against consent as a default justification for collecting data from learning activities?

In terms of legitimate interests, Cormack (2016a) sums up how European law specifies requirements for this justification to be used:

Where personal data are processed for legitimate interests, there must be a clearly stated purpose, the processing must be necessary for that purpose, the impact and risk for the individuals whose data are processed must be minimised, and any remaining impact or risk must be justified by a balancing test against the claimed interest. Interests, even though legitimate, cannot justify processing that involves an inappropriate risk to the individuals whose data are processed. (Cormack 2016a).

The schools and universities need to know what they want to achieve with data analysis, otherwise they do not pass the ‘necessity test’: information that is not necessary for the declared purposes should not be collected (Cormack 2016b). And there is no way out for the institutions to turn to the students and ask for a blanket acceptance of collecting data. The students need to know what they are asked about, to be able to balance the benefits and risks of the proposal. Data-driven techniques, not guided by questions or hypothesis, where the ideas of possible interventions first appear after the data are collected and processed do not give much in terms of specific purpose descriptions for justifying the process before it is started.

The students need to be actively involved, as we see when LA is set up to personalise learning. Cormack makes it clear that legitimate interests cannot be used to justify any activity where the intention is to personalise a service or otherwise affect individual users, ‘since this would contradict the requirement that the impact on individuals be minimised’ (Cormack 2016a).

Once the organisation has identified patterns in data that enable it to identify and design such an intervention, however, it should also have sufficient information to seek valid consent from those individuals who may be affected by it. Whereas at the time the data were collected the results of data-driven analysis and their consequences could not be foreseen or explained to individuals, now they can. Consent can now be fully informed. Offering a choice between personalised and generic versions of the service should increase the likelihood that consent to personalisation is freely given. (Cormack 2016a).

The constraints of the law and the intrinsic qualities of data-driven practices that LA is part of seem to drive LA implementers towards what Sclater (2017) has called a hybrid approach: using *legitimate interests for analysis and consent for intervention*. Cormack (2016a, 2016b) has argued that the solution, which came up in the discussion of consent related to the developed of GDPR, termed ‘downstream’ consent should be applied: ‘consent can also be requested “downstream”, when the purpose of the processing changes’ (Article 29 Data Protection Working Party 2011). Upstream there is the analysis of the data, trying to identify patterns; downstream are the interventions to be taken when one knows what the problem is, it is still not acted upon, and one is able to communicate clearly to the student options that the student could agree to.

The approach proposed by Cormack (2016a, 2016b) dividing the monolithic ‘big data’ process into two stages (analysis, to find patterns; and intervention, to identify and affect relevant individuals) opens up a need for examining the educational specific consequences and opportunities when applied on LA. This is the focus of the last part of this paper.

## Pedagogical opportunities arising from LA data privacy

In theory, a separation of LA into two processes, analysis and intervention seems simple. Analysis justified by legitimate interests is the prerogative of the institution; students are first involved when clear actions can be outlined with opt-in and opt-out options to consent to. To perceive this as two distinct processes with no overlapping stakeholders and no interfering sub-processes seems, however, to be too far from real life in education. What about the teachers, are they part of the analysis process? What about access to data? Does data for analysis come only from institutional systems, like Student Information Systems or Learning Management Systems? How does the institution get access to data from non-institutional and informal learning settings, e.g. mobile and cloud learning platforms outside the control of the school or university, social media, other sensor data relevant for learning?

**Fig. 4:**
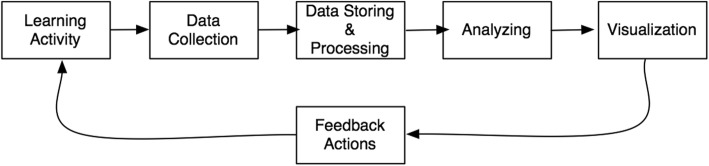
LA process model developed by ISO/IEC JTC1/SC36 (ISO 2016)

Contrasting the hybrid model of analysis and intervention with the LA process model developed by ISO/IEC JTC1/SC36 (Fig. 4), we see that three important sub-processes precede the analysis stage. In order to be able to do analysis one needs to decide upon which learning activities to monitor; to collect the data that serve as proxies for the activities under study; and as a last important step also imbued with a host of privacy and data protection issues, decide upon how the data should be stored and processed before analysis.

It feels strange to exclude these introductory processes from exchange with the students under the pretext that the analysis of these activities is within the legitimate interest of the institution. On the other hand, it might well be that conversations about what is going on prior to and during analysis are part of activities that are crossing different professional and educational discourses with associated norm sets. Learning analytics is different from traditional academic analytics, which does not aim at actionable insights feeding back to the individual learner (Gasevic et al. 2015). Therefore, analysis cannot only be an administrative task, or a pure research activity. And with teachers on board doing analysis, this is definitely also a pedagogical activity, which involves the learners. To see how it involves learners, and how it is different from intervention, we first need to look at what characterises intervention.

It is the risks to the learner, caused by the institution acting upon the knowledge from analysis that make it necessary to ask the learner to consent to processing of personal data, giving him or her the opportunity to opt out when the nature of the proposed intervention is clarified. Even if these deliberations have a legal flair to them, they are mainly of pedagogical nature. The worst scenario from a student’s perspective is probably illegal: that predictive profiling could be subject to automated processing leading for example to exclusion (Hoel and Chen 2016). Most likely, interventions would be to present the learner with different alerts and prods (e-mails or messages); visualisations showing progress, position relative to different student cohorts, etc.; and recommendations for what to read next, what tests to take next, etc. Some of these interventions will be executed by machine, but most likely the majority will involve interaction between the students and the teachers.

A comparative case study of educational big data practices in Norway and China (Hoel et al. 2018) substantiates that the pedagogical reality in some cultures may prove difficult to fit within strict legal schemas of what data could or should be used for analytics. In Tongzhou, a district of Beijing, we found that teachers had an almost unlimited appetite for information about their students. In some instances, they also had instruments to gather data for psychological profiling that was not directly related to specific learning activities or subject areas. The contrast to Norwegian primary and secondary education was sharp, as the Oslo teachers expressed interest primarily in information about knowledge acquisition and subject-related issues in school. Off school activities, use of social media, family relations, etc. were not something Oslo teachers wanted to gather data about on a regular basis. Both the Tongzhou and the Oslo teachers had strong pedagogical motivation for their interest in student activity data.

In conclusion, looking back to the LA process model (Fig. 4), both analysis (and the preceding sub-processes) and intervention will involve extensive interaction with the students. We have difficulties seeing that questions of data access and handling are dealt with inside a secluded administrative and research logic without involvement of students and teachers, and their virtual learning agents. That being said, we see the value of keeping the separation between analytical and intervention concerns, being forced to execute the balancing test, weighing the benefits and risks of collecting and processing personal data. We believe that different normative models could live side by side. Table 2 summarises the focus and questions of the different models governing data handling.

**Table 2:** Models for handling data in educational setting

Model for data handling	Model focus	Question asked
Legal model	Justified purpose for data collection?	Are the risks to the individual balanced with the benefits to the individual and the system?
Research model	Consent, fair data handling, and safe data keeping	Have participants agreed to be part of the research?
Administrative model	Handling of personally identifiable information	Are the data de-identified and kept safe?
Pedagogical model	Learning gain	Are collected data relevant for understanding and optimising learning and the environments in which it occurs?

The legal model tells you to wait to ask for consent until the individual has a chance to make an informed choice based on alternative proposals for intervention. The research model tells you to ask for permission to gather information, to follow the fair processing principles and to keep the data safe. The administrative model tells you to use anonymised aggregated data and follow strict legal procedures when dealing with personal information. Most importantly, the pedagogical model tells you to support the student’s own learning and use every opportunity to enhance the learning experience by bringing in relevant tasks and material. Data for learning analytics is as such an opportunity to enhance students’ data literacy.

## Conclusions and future work

We introduced this study with the challenges faced by an international group of standards experts trying to motivate global norms for privacy and data protection in the context of learning analytics. How do we find a common ground for policy development when we have countries where all learning activity data seem to be available for analysis (e.g. China), and countries that are reluctant to allow library data to be analysed because of privacy issues, and questions whether learning analytics is legal in the first place (e.g. Norway) (Hoel et al. 2018; Hoel and Chen 2017; Hoel et al. 2017)? It would help to build consensus about privacy and data protection policies if these also could be argued from an educational perspective, not only from universal or individual rights perspectives.

In this paper we have demonstrated that privacy as a ‘hypernorm’ yields when pressured by corporate, commercial, or national security interests. Likewise, consent as general justification for collection and processing of personal data is not applicable in an educational setting unless the process is carefully staged, separating analysis from intervention. The discussion of justifications for accessing and processing learning activity data has shown that we from the very beginning are within a space of negotiations, using a variety of justifications based on ethics, law, national policies, and pedagogies. Therefore, we should in LA make an effort of making the pedagogical justification for privacy policies more explicit.

This result of our explorations, the positioning of privacy and data protection for LA in an argumentative space of continuous negotiations, gives hope for achieving an international consensus on educational privacy policies. From our discussion in this paper the following principles emerge as starting point for further development:

1. Privacy and data protection in LA are achieved by negotiating data sharing with each student.
2. Openness and transparency are essential and should be an integral part of institutional policies. How the educational institution will use data and act upon the insights of analysis should be clarified in close dialogue with the students.
3. Big data will impact all society. Therefore, in negotiating privacy and data protection measures with students schools and universities should use this opportunity to strengthen their personal data literacies.

These principles have strong grounding in discourse and practice on privacy. The first principle is in accord with the theory of contextual integrity proposed by Nissenbaum (2014).

The theory of contextual integrity is a theory of privacy with respect to personal information because it posits that informational norms model privacy expectations; it asserts that when we find people reacting with surprise, annoyance, indignation, and protest that their privacy has been compromised, we will find that informational norms have been contravened, that contextual integrity has been violated. (Nissenbaum 2014, p. 25).

The second principle is in accordance with the best practice guidelines we now see published by educational institutions informing about how LA will be implemented (Sclater 2016; Open University UK 2014).

The third principle connects to the discussion on twenty-first century skills and competences for new millennium learners (Ananiadou and Claro 2009). In Norwegian education, for more than a decade digital literacy has been defined as one of the central competences needed in the future, and the ability to use digital tools was defined as a basic skill (Sefton-Green et al. 2009; Krumsvik 2008, 2009). Understanding how student data are used is part of digital literacy (Pangrazio and Selwyn 2018).

We would suggest that these principles are further developed and expressed in a LA privacy maxim for education. The conceptual and explorative research presented in this paper has limitations as expected when addressing a new field of enquiry. We will therefore follow up this research with more empirical studies of how different countries develop privacy policies in education, and how we can develop solutions for privacy in LA that could be accepted across cultures. This paper has shown that there is a need to understand how data privacy policies for LA connect to pedagogical practices. How future design of LA tools could use this educational argumentative space will be subject of further studies.

## Abbreviations

GDPR: General Data Protection Regulation
LA: Learning analytics

## References

[CR1] Ananiadou K, Claro M (2009). 21st Century Skills and Competences for New Millennium Learners in OECD Countries (Vol. 41).

[CR2] Article 29 Data Protection Working Party, Opinion 15/ 2011 on the definition of consent (01197/11/EN WP 187) 19.

[CR3] Bygrave, L. A. (2010). Privacy and data protection in an international perspective. Scandinavian Studies in Law. Online: http://www.scandinavianlaw.se/pdf/56-8.pdf, http://www.scandinavianlaw.se/pdf/56-8.pdf. Accessed 2 Aug 2017.

[CR4] Chen, L.Y. (2018). Jack Ma's ant apologizes for baiting users into credit system. Bloomberg. 4 January 2018. https://www.bloomberg.com/news/articles/2018-01-04/jack-ma-s-ant-apologizes-for-baiting-users-into-credit-system

[CR5] Cormack, A. (2016b). A data protection framework for learning analytics. *Journal of Learning Analytics*, 91–106 10.18608/jla.2016.31.6.

[CR6] Cormack, A. N. (2016a). Downstream consent: A better legal framework for big data. *Journal of Information Rights, Policy and Practice, 1*(1). 10.21039/irpandp.v1i1.9.

[CR7] Datatilsynet. (2013). Big Data – privacy principles under pressure. September 2013. Online: https://www.datatilsynet.no/globalassets/global/english/big-data-engelsk-web.pdf. Accessed 24 July 2017.

[CR8] European Commission. (2016). REGULATION (EU) 2016/679 OF THE EUROPEAN PARLIAMENT AND OF THE COUNCIL of 27 April 2016 on THE protection of natural persons with regard to the processing of personal data and on the free movement of such data, and repealing directive 95/46/EC (general data protection Regulation).

[CR9] Facebook–Cambridge Analytica data scandal. (n.d.). Retrieved 21 August 2018 from https://en.wikipedia.org/wiki/Facebook%2DCambridge_Analytica_data_scandal

[CR10] Gasevic D, Dawson S, Siemens G (2015). Let’s not forget: Learning analytics are about learning. TechTrends.

[CR11] Hazari S, Brown C (2014). An empirical investigation of privacy awareness and concerns on social networking sites. Journal of Information Privacy and Security.

[CR12] Hoel, T., & Chen, W. (2016). Implications of the European data protection regulations for learning analytics design. Workshop paper presented at the international workshop on learning analytics and educational data mining (LAEDM 2016) in conjunction with the international conference on collaboration technologies (CollabTech 2016), Kanazawa, Japan - September 14–16, 2016. Retrieved March 24, 2017, from http://hoel.nu/files/LAEDM_Kanazawa_Sep2016_Hoel_Chen_final_w_header.pdf

[CR13] Hoel T, Chen W (2017). Innovating Learning Analytics Policies in Norway - a case study. Draft workshop paper presented at LAK17 workshop on LA Policies.

[CR14] Hoel, T., Chen, W., & Gregersen, A. B. (2018). Are Norwegian librarians ready to share library data to improve learning? *Nordic Journal of Information Literacy in Higher Education, 10*(1). 10.15845/noril.v10i1.269.

[CR15] Hoel T, Chen W, Griffiths D (2017). Is international consensus about privacy policies for learning analytics possible? Draft workshop paper presented at LAK17 workshop on LA policies.

[CR16] Hoel T, Chen W, Yu L (2018). Is big data going to change education? A comparative case study from Norway and China. Manuscript submitted for publication.

[CR17] Hoel T, Griffiths D, Chen W (2017). The influence of data protection and privacy frameworks on the design of learning analytics systems (pp. 243–252). Presented at the the Seventh International Learning Analytics & Knowledge Conference, New York.

[CR18] ISO. (2016). ISO/IEC TR 20748–1:2016 Information technology for learning, Education and Training -- Learning analytics interoperability -- Part 1: Reference model.

[CR19] Krumsvik R (2008). Situated learning and teachers’ digital competence. Education & Information Technologies.

[CR20] Krumsvik R (2009). Situated learning in the networked society and the digitised school. European Journal of Teacher Education.

[CR21] MEITY. (2018). THE PERSONAL DATA PROTECTION BILL, 2018. Ministry of Electronics and Information Technology, India. Online: http://meity.gov.in/content/personal-data-protection-bill-2018

[CR22] Milberg, S. J., Burke, S. J., Smith, H. J., & Kallman, E. A. (1995). Values, personal information privacy, and regulatory approaches. Communications of the ACM, 38(12), 65–74. 10.1145/219663.219683

[CR23] Milberg SJ, Smith HJ, Burke SJ (2000). Information privacy: Corporate management and National Regulation. Organization Science.

[CR24] Nissenbaum, H. (2014). Respect for Context as a Benchmark for Privacy Online: What It Is and Isn’t. In “The Future of Privacy”, Foundation Télécom, Institute Mines-Télécom, 1–128. Online: http://cvpip.wp.mines-telecom.fr/files/2014/02/14-02-The-futur-of-privacy-cahier-de-prospective.pdf. Accessed 8 Aug 2017.

[CR25] Open University UK (2014). Policy on Ethical use of Student Data for Learning Analytics. Online: https://help.open.ac.uk/documents/policies/ethical-use-of-student-data/files/22/ethical-use-of-student-data-policy.pdf. Accessed 9 Aug 2017.

[CR26] Pangrazio L, Selwyn N (2018). “Personal data literacies”: A critical literacies approach to enhancing understandings of personal digital data. New Media & Society.

[CR27] Piper DLA (2017). Data Protection Laws of the World.

[CR28] Sclater, N. (2016). Developing a code of practice for learning analytics. *Journal of Learning Analytics*, 16–42 10.18608/jla.2016.31.3.

[CR29] Sclater, N. (2017). Consent for learning analytics: some practical guidance for institutions. Blog post, February 16, 2017. Online: https://analytics.jiscinvolve.org/wp/2017/02/16/consent-for-learning-analytics-some-practical-guidance-for-institutions.

[CR30] Sefton-Green J, Nixon H, Erstad O (2009). Reviewing approaches and perspectives on “digital literacy”. Pedagogies.

[CR31] Elana Zeide (2017), The Limits of Education Purpose Limitations, 71 U. Miami L. Rev. 493 Online: http://repository.law.miami.edu/umlr/vol71/iss2/8. Accessed 8 Aug 2017.

